# UAV Array-Aided Visible Light Communication with Enhanced Angle Diversity Transmitter

**DOI:** 10.3390/s25185752

**Published:** 2025-09-15

**Authors:** Weiren Wang, Zhihong Zeng, Chen Chen, Dengke Wang, Min Liu, Harald Haas

**Affiliations:** 1 School of Microelectronics and Communication Engineering, Chongqing University, Chongqing 400044, China; 20231201022g@cqu.edu.cn (W.W.); dengke.wang@cqu.edu.cn (D.W.); liumin@cqu.edu.cn (M.L.); 2 Department of Engineering, University of Cambridge, 9 JJ Thomson Avenue, Cambridge CB3 0FA, UK; huh21@cam.ac.uk

**Keywords:** visible light communication (VLC), unmanned aerial vehicle (UAV), enhanced angle diversity transmitter (ADT)

## Abstract

Visible light communication (VLC) aided by unmanned aerial vehicles (UAVs) offers significant advantages in adapting to dynamic network requirements, but the endurance and service capability of UAVs are still the key limiting factors. To overcome this limitation, the UAV array-aided VLC system with an enhanced angle diversity transmitter (ADT) is proposed to improve energy efficiency (EE). Enhanced ADTs with varying LED layers, multiple LEDs per layer, and inter-layer rotation angles are considered. By jointly optimizing the inclination angle of the side LEDs in the enhanced ADT and the hovering height of the UAVs, this research aims to minimize the power consumption of the UAV array-aided VLC system while meeting illumination and communication requirements. The simulation results present that the EE of the centralized single-UAV VLC system can be greatly improved by applying the enhanced ADT structures. More specifically, compared with the single LED transmitter configuration, an EE enhancement of up to 215.7% can be achieved by the enhanced ADT, which employs multi-layer LEDs, inter-layer rotation, and layer-doubled designs. In addition, the EE can be further improved by the deployment of a distributed UAV array. The VLC system with four UAVs is demonstrated to achieve a peak EE performance of 19.9 bits/J/Hz, representing a 298% improvement over the centralized single-UAV configuration.

## 1. Introduction

The use of unmanned aerial vehicles (UAVs) as airborne base stations presents a versatile and economical solution to provide adaptive communication services [1,2,3,4]. Nevertheless, in forthcoming ultra-dense wireless networks, conventional RF-based aerial base stations may generate substantial interference with terrestrial devices, potentially degrading ground network performance. Furthermore, energy constraints pose significant limitations on the ability of UAVs to deliver high-speed RF communication services effectively. These limitations can be overcome through the integration of visible light communication (VLC) [4,5,6,7,8,9,10]. VLC technology has gained considerable interest in recent years owing to its expansive unregulated spectrum and superior power efficiency. Light-emitting diode (LED)-based VLC systems, for example, offer dual functionality by combining illumination with data transmission capabilities [11,12,13]. The aerial positioning of UAVs naturally establishes line-of-sight (LOS) channels ideal for VLC implementation, making this approach particularly suitable for bandwidth-intensive and energy-conscious UAV communication systems. Due to their flexible mobility and adaptive altitude control [3], UAV-VLC systems are especially well-suited for applications such as nighttime search and rescue operations, disaster response, and other scenarios that demand simultaneous emergency illumination and communication capabilities [14]. However, the practical deployment of such systems is still hampered by limited energy capacity, which significantly restricts their operational endurance [15].

Previous studies have explored various methods to enhance the endurance and service capabilities of UAVs [16,17,18]. In [16], the authors investigated a joint optimization of power allocation and UAV positioning to maximize the sum rate of all users, subject to constraints on transmit power, quality-of-service requirements, and UAV mobility. The authors in [17] jointly optimized the UAV deployment, user association, and power efficiency to meet emergency illumination and communication demands. Furthermore, a novel framework for UAV trajectory planning and coverage control is proposed to enhance monitoring capabilities [18]. These approaches, which integrate VLC with UAVs using LEDs, offer flexible solutions for emergency illumination and communication scenarios. However, existing studies have relied mainly on simple LED transmitters for dual illumination-communication functionality, leaving more advanced transmitter designs unexplored in UAV-VLC systems.

Angle diversity techniques have been shown to significantly improve the performance of the VLC system. At the receiver side, employing an angle diversity receiver (ADR) with appropriate combining schemes can improve both communication rates and overall system performance [19,20,21,22,23]. Moreover, implementing an angle diversity transmitter (ADT) at the transmitter side has demonstrated performance benefits in VLC systems [24]. However, the application of ADT in UAV-VLC systems has not yet been studied. Therefore, our prior work in [25] introduced and evaluated a two-layer ADT for UAV-assisted VLC downlink systems. While this previous study demonstrated the potential of two-layer transmitter designs, it was limited to single-UAV configurations, which may prove inadequate for large-scale coverage scenarios. Furthermore, the investigation did not explore optimized ADT configurations incorporating multiple layers or advanced structural designs, which could potentially enhance both the energy efficiency (EE) and coverage performance of the system. These limitations motivate the current work to examine more advanced ADT architectures in multi-UAV array configurations.

To address the above challenges, this paper proposes two key innovations: (1) enhanced ADT designs for centralized single-UAV VLC downlink systems; and (2) distributed UAV array architectures where multiple UAVs are equipped with enhanced ADTs. The proposed enhanced ADT optimizes both the UAV hover height and transmitter inclination angle to minimize power consumption while satisfying user illumination and communication requirements, thereby maximizing the EE of the system. Simulations are conducted to evaluate the performance of UAV array-aided VLC downlink systems with enhanced ADTs. It is shown that the distributed UAV array-aided VLC systems outperform the centralized single-UAV VLC systems in terms of coverage and EE.

## 2. System Model

**Figure 1:**
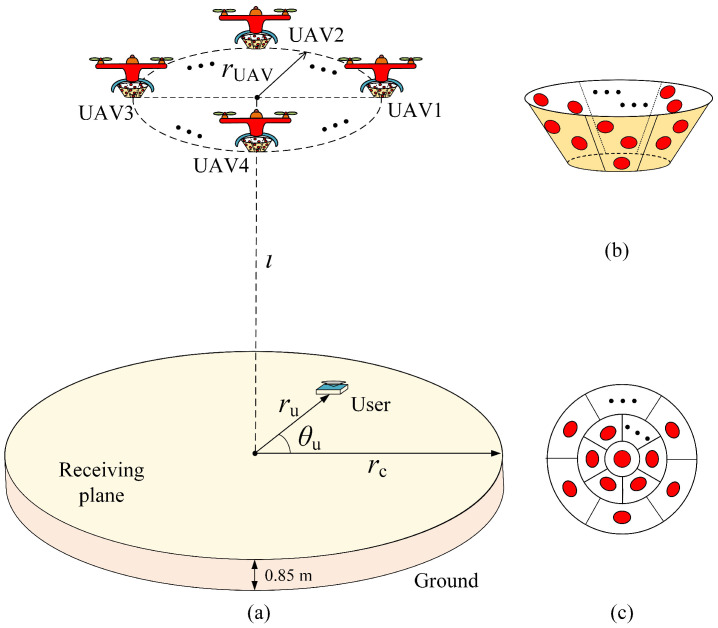
(**a**) System model of UAVs-VLC using enhanced ADT, (**b**) geometric structure of enhanced ADT, and (**c**) bottom view of enhanced ADT.

This paper examines two distinct system architectures for UAV-VLC systems: the centralized single-UAV configuration and the distributed UAV-array configuration. In both systems, each UAV is equipped with ADTs capable of providing integrated illumination and communication services to users within the coverage area. Figure 1a illustrates the fundamental model of the distributed UAV-array aided VLC systems. The system comprises a distributed array of UAVs operating on a shared horizontal plane at a fixed altitude *l* above the receiving plane. The UAV array collaboratively provides coverage for a circular target region with radius $r_{c}$. Meanwhile, the user’s location is specified in polar coordinates as $\left(r_{u},\theta_{u}\right)$, where $r_{u}$ represents the radial distance from the origin and $\theta_{u}$ denotes the angular position relative to the reference axis. Moreover, the ADT designed for the UAV-VLC system consists of totally *N* LEDs, including one bottom LED and N−1 inclined side LEDs. The geometric structure of the ADT is illustrated in Figure 1b while the bottom view of the ADT is shown in Figure 1c. In the centralized single-UAV configuration, the UAV array shown in Figure 1a is replaced by a centralized single UAV.

**Figure 2:**
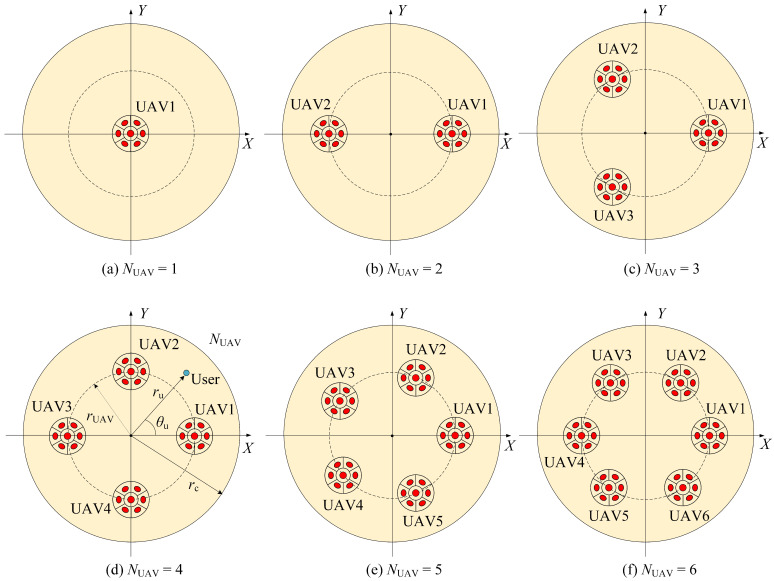
Top view of UAV array distribution.

The number of UAVs in the UAV array is denoted by $N_{\mathrm{UAV}}$. Figure 2a–f presents the top view of the UAV-array configuration corresponding to $N_{\mathrm{UAV}}$ ranging from 1 to 6. The red dots represent the transmitters mounted on UAVs, and the yellow circle with radius $r_{c}$ represents the target coverage area. For $N_{\mathrm{UAV}}\geq 2$, the UAVs are assumed to be uniformly deployed on a circle of radius $r_{\mathrm{UAV}}$ concentric with the target receiving plane. The system configuration is determined by the number of UAVs, where $N_{\mathrm{UAV}}=1$ yields the centralized single-UAV case, while $N_{\mathrm{UAV}}\geq 2$ results in distributed UAV-array configurations.

### 2.1. The Structure of the Enhanced ADT

**Figure 3:**
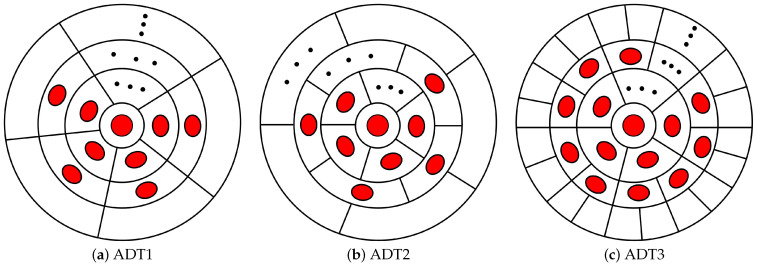
Bottom view of different types of enhanced ADTs.

As shown in Figure 3, the enhanced ADT is composed of *N* LEDs, including one LED at the bottom center position and N−1 LEDs at the side slope. To be more specific, the LEDs are separated into *K* layers and the number of LEDs in the *k*-th layer is denoted as $n_{k}$. Therefore, $N=\sum_{1}^{K}n_{k}$ and $n_{1}=1$. Here, it is assumed that all LEDs in the enhanced ADT have the same optical and electrical properties. In this paper, we propose three types of enhanced ADTs, namely, ADT1, ADT2, and ADT3. The bottom views of the proposed enhanced ADTs are illustrated in Figure 3. Specifically, ADT1 represents the basic multi-layer structure, ADT2 extends this design by introducing inter-layer rotation, and ADT3 further enhances it through a layer-doubling architecture combined with inter-layer rotation. A detailed description of the three proposed enhanced ADTs is provided below.

**Figure 4:**
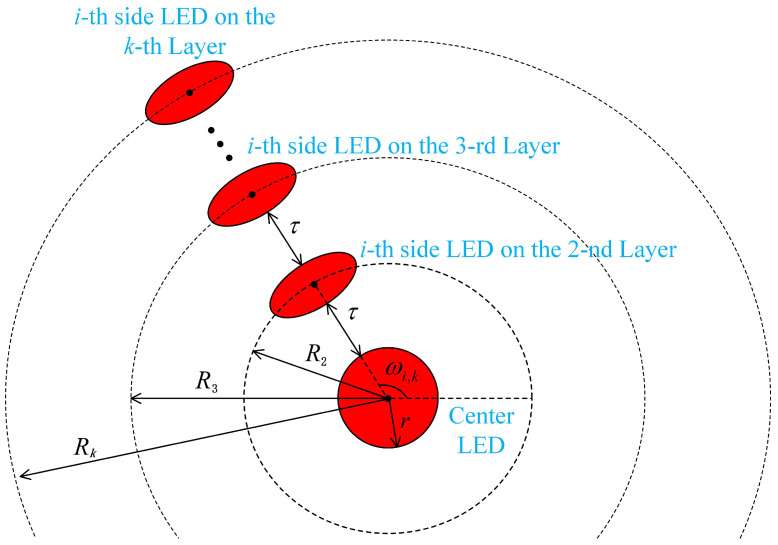
Top view of the enhanced ADT structure.

The ADT1 is constructed through the sequential addition of concentric layers comprising side-inclined LEDs, with each outer layer maintaining identical azimuthal spacing while replicating the geometric configuration of its adjacent inner layer, as illustrated in Figure 3a. The bottom view of ADT1 configuration presented in Figure 4 clearly demonstrates the positional structure and geometric relationships among the *i*-th LEDs across different layers. Specifically, the first-layer LED is positioned at the bottom center with radius *r*, while the *i*-th side-inclined LEDs in the second, third, and *k*-th layers are arranged on concentric circles with radii $R_{2}$, $R_{3}$, and $R_{k}$, respectively. A uniform inter-layer spacing τ is maintained between adjacent layers throughout the configuration. The azimuth angle of the *i*-th LED in the *k*-th layer is denoted as $\omega_{i,k}^{\mathrm{ADT}1}$. Assuming that the azimuth angle of the first side-tilting LED in *k*-th layer is fixed at $\omega_{1,k}^{\mathrm{ADT}1}=0{}^{\circ}$, the azimuth angle of the *i*-th LED in *k*-th layer for ADT1 can be obtained as follows:

(1) $$\omega_{i,k}^{\mathrm{ADT}1}=360{}^{\circ}\times \frac{i-1}{n_{k}},\;k>1.$$

As each outer layer of ADT1 maintains an identical number of LEDs, the number of LEDs in the *k*-th layer can be expressed as $n_{k}=\frac{N-1}{K-1}$. Therefore, $\omega_{i,k}^{\mathrm{ADT}1}$ in (1) can be rewritten as

(2) $$\omega_{i,k}^{\mathrm{ADT}1}=360{}^{\circ}\times \frac{\left(i-1)(K-1\right)}{N-1},\;k>1.$$

It should be noted that in ADT1, the *i*-th azimuth angle of the LEDs remains constant across different layers, as expressed in (2), since this parameter is independent of the layer index *k*. This characteristic ensures angular alignment of corresponding LEDs throughout the layered structure.

The ADT2 configuration, shown in Figure 3b, preserves the core layered architecture of ADT1 in Figure 3a while introducing a inter-layer azimuth rotation by angle β between corresponding side-tilted LEDs in adjacent layers. This modification yields the following expression for the azimuth angle of the *i*-th LED in the *k*-th layer:

(3) $$\omega_{i,k}^{\mathrm{ADT}2}=360{}^{\circ}\times \frac{\left(i-1)(K-1\right)}{N-1}+\left(k-2\right)\beta ,\;k>1.$$

Figure 3c illustrates the ADT3 configuration, which incorporates two key design modifications relative to the designs of ADT1: (1) a doubling of LED count between consecutive layers $\left(n_{k}=2n_{k-1},k>2\right)$, and (2) the introduction of a systematic inter-layer azimuthal rotation β. Therefore, the azimuth angle of the *i*-th LED in *k*-th layer for ADT3 can be obtained as

(4) $$\omega_{i,k}^{\mathrm{ADT}1}=360{}^{\circ}\times \frac{i-1}{n_{k}}+\left(k-2\right)\beta ,\;k>1.$$

This results in an exponentially increasing LED density with layer depth while maintaining precise angular control through the β rotation parameter.

### 2.2. Optical Channel Gain

For the outdoor VLC system under consideration, we make the following fundamental assumptions: First, the channel is dominated by LOS propagation, allowing non-line-of-sight (NLOS) components to be neglected due to their minimal contribution in typical outdoor environments; second, the UAV maintains perfect horizontal hovering with its bottom-mounted LEDs of the ADT oriented vertically downward while side-mounted LEDs maintain their designated inclination angles; third, the photo-detector (PD) of the user is vertically aligned (normal to the ground plane); fourth, each LED mounted in the ADT follows the Lambertian radiation patten.

Let $N_{\mathrm{UE}}$ denote the total number of possible user locations within the coverage of the system, with the complete set of user positions defined as $U=\{j|j\in \left[1,N_{\mathrm{UE}}\right]\}$. For the centralized single-UAV configuration, the LOS optical channel gain between the *i*-th side-inclined LED in the *k*-th layer of the ADT and the PD at the *j*-th user location can be expressed as [25]

(5) $$h_{i,k,j}=\frac{(m+1)A}{2\pi d_{i,k,j}^{2}}\cos^{m}\left(\theta_{i,k,j}\right)g_{f}g_{c}\cos\left(\gamma_{i,k,j}\right),$$

where m=−ln2/lncosΦ is the order of Lambertian emission, with Φ being the semi-angle at half power of each LED; *A* is the receiving area of the PD; $d_{i,k,j}$ is the distance between the *i*-th LED in the *k*-th layer of the ADT and the PD at the *j*-th user location, where $\theta_{i,k,j}$ and $\gamma_{i,k,j}$ are the corresponding emission angle and incidence angle, respectively; $g_{f}$ is the gain of the optical filter; and $g_{c}=\frac{n^{2}}{\sin^{2}\Psi }$ is the gain of the optical concentrator with *n* and Ψ, respectively, denoting the refractive index and the field-of-view (FOV) of the optical concentrator. Each enhanced ADT comprises *K* concentric LED layers, with the *k*-th layer containing $n_{k}$ LEDs. Given that all LEDs in the ADT transmit identical signals, the aggregate optical channel gain $H_{j}^{\mathrm{Central}}$ at the *j*-th user location for the centralized single-UAV configuration is given by

(6) $$H_{j}^{\mathrm{Central}}=\sum_{k=1}^{K}\sum_{i=1}^{n_{k}}h_{i,k,j}.$$

For the distributed UAV-array configuration, it is assumed that there are $N_{\mathrm{UAV}}$ UAVs equipped with the same enhanced ADTs to provide illumination and communication services to users in the target area, and that all LEDs in the enhanced ADTs transmit the same electrical signal. Therefore, for the *a*-th UAV, the LOS optical channel gain between the *i*-th side-inclined LED in the *k*-th layer of the ADT and the PD at the *j*-th user location can be expressed as

(7) $$h_{a,i,k,j}=\frac{(m+1)A}{2\pi d_{a,i,k,j}^{2}}\cos^{m}\left(\theta_{a,i,k,j}\right)g_{f}g_{c}\cos\left(\gamma_{a,i,k,j}\right),$$

where $d_{a,i,k,j}$ is the distance between the *i*-th LED in the *k*-th layer of the ADT on *a*-th UAV and the PD at the *j*-th user location, where $\theta_{a,i,k,j}$ and $\gamma_{a,i,k,j}$ are the corresponding emission angle and incidence angle, respectively. Therefore, the aggregate optical channel gain $H_{j}^{\mathrm{Distributed}}$ at the *j*-th user location for the distributed UAV-array configuration is given by

(8) $$H_{j}^{\mathrm{Distributed}}=\sum_{a=1}^{N_{\mathrm{UAV}}}\sum_{k=1}^{K}\sum_{i=1}^{n_{k}}h_{a,i,k,j}.$$

### 2.3. Energy Efficiency

The limited onboard energy capacity of UAVs makes EE optimization crucial for UAV-assisted VLC systems, particularly when maintaining both illumination and emergency communication requirements. This study specifically aims to minimize the system’s power consumption by jointly optimizing two key parameters: (1) the hovering altitude of UAVs *l* and (2) the inclination angles φ of the side-mounted LEDs in the ADT. Our analysis focuses exclusively on the power consumption of the LED-based illumination and communication systems. The achievable data rate per unit bandwidth for the user at the *j*-th position can be approximated by [26]

(9) $$C_{j}=\frac{1}{2}\log_{2}\left(1+\frac{e}{2\pi }{\left(\frac{\varepsilon P_{j}H_{j}}{\sigma_{w}}\right)}^{2}\right),$$

where ε denotes the illumination target [27], $P_{j}$ represents the optical power emitted by the UAV, and $\sigma_{w}$ is the standard deviation of the additive white Gaussian noise. Given a data rate threshold $C_{\mathrm{th}}$ required for the user to establish a successful communication link, the minimum optical power needed to satisfy the communication requirement at the *j*-th user position can be derived from (9) as

(10) $$P_{j,\min}^{\mathrm{Communication}}=\frac{\sigma_{w}\sqrt{\frac{2\pi }{e}\left(2^{2C_{\mathrm{th}}}-1\right)}}{\varepsilon H_{j}}.$$

Moreover, the illuminance at the *j*-th user position can be calculated by [28]

(11) $$\kappa_{j}=\varepsilon P_{j}^{\mathrm{Illumination}}H_{j},$$

where $H_{j}$ for the single-UAV configuration and the distributed UAV-array configuration can be derived from (6) and (8), respectively. Letting $\kappa_{\mathrm{th}}$ represent the illuminance threshold for effective illumination, the minimum required optical power at the *j*-th user position to satisfy the illumination constraint can be derived from (11) as

(12) $$P_{j,\min}^{\mathrm{Illumination}}=\frac{\kappa_{\mathrm{th}}}{\varepsilon H_{j}}$$

To satisfy the joint communication and illumination requirements across all potential user locations, the minimum required optical power can be derived as

(13) $$P_{\min}=\max_{j\in U}\left\{P_{j,\min}^{\mathrm{Communication}},P_{j,\min}^{\mathrm{Illumination}}\right\},$$

which corresponds to the $j^{*}$-th user position. Therefore, the corresponding EE of the UAV-VLC system can be obtained as follows:

(14) $$\eta =\frac{C_{j^{*}}}{P_{\min}}=\frac{1}{2P_{\min}}\log_{2}\left(1+\frac{e}{2\pi }{\left(\frac{\varepsilon P_{\min}H_{j^{*}}}{\sigma_{w}}\right)}^{2}\right),$$

where $C_{j^{*}}$ denotes the achievable rate of the user at the $j^{*}$-th position.

## 3. Simulation Results

Section 3 evaluates the EE of the enhanced ADT-assisted UAV-VLC system in a practical deployment scenario combining illumination and communication functionalities. The system configuration employs enhanced ADTs with LEDs characterized by a semi-angle at half power of Φ=30°. The geometric arrangement consists of a receiver plane positioned at 0.85 m above ground level, utilizing LEDs with a radius of r=5 mm and maintaining a inter-layer spacing τ of 5 cm. These parameters form the baseline scenario for evaluating energy efficiency (EE). To enable joint emergency illumination and communication, the channel capacity threshold is specified as 1 bit/s/Hz and the illuminance threshold is assumed to be $5\times 10^{-4}$ Lx [16,17,27]. Additional simulation parameters are provided in Table 1. All simulations are implemented in MATLAB R2016a.

### 3.1. The Centralized Single-UAV Configuration

This study first conducts a comparative performance analysis of multiple ADT configurations implemented in a centralized single-UAV VLC system. The investigation evaluates three distinct ADT architectures (ADT1, ADT2, and ADT3) with varying LED distribution patterns and rotational characteristics to determine their impact on system perform- ance metrics.

**Figure 5:**
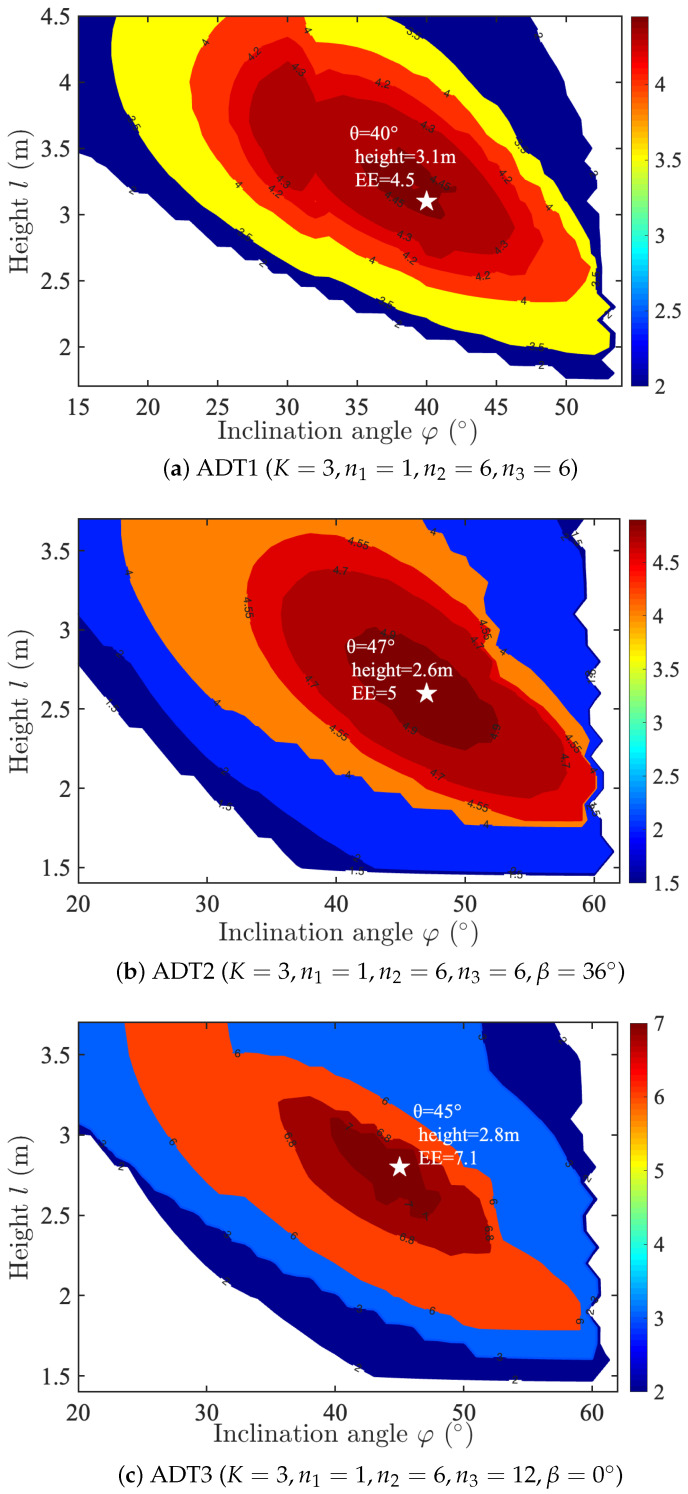
Contour plot of the EE (bits/J/Hz) for different ADT configurations in the centralized single-UAV VLC system.

Figure 5a demonstrates the optimized EE landscape for the ADT1 configuration, where the EE of the centralized single-UAV VLC system depends on both the LED inclination angle φ and UAV hover height *l*. The analysis considers a three-layer ADT1 structure (K=3) with $\left[n_{1},n_{2},n_{3}\right]=\left[1,6,6\right]$ LEDs per layer and a target coverage area of radius $r_{c}=3$ m. The contour plot reveals a distinct global maximum EE of 4.5 bits/J/Hz (marked by a white star) achieved at φ=40° and l=3.1 m. Parameter combinations in white regions fail to satisfy the dual requirements of emergency communication and illumination, a convention consistently applied in subsequent analyses.

Figure 5b presents the optimized EE contour plot (in bits/J/Hz) for the ADT2 in a centralized single-UAV VLC system, demonstrating the joint influence of the LED inclination angle φ and UAV hover height *l* under the following parameterization: (1) K=3 layers with $n_{1}=1$, $n_{2}=6$, and $n_{3}=6$ LEDs per layer, and inter-layer rotation angle β=36°; (2) target area radius $r_{c}=3$ m. The analysis reveals an optimal operating point achieving peak EE of 5.0 bits/J/Hz at φ=47° and l=2.6 m, representing an 11.12% improvement over the ADT1 configuration in Figure 5a. These results clearly demonstrate the performance advantage of incorporating inter-layer rotation β in the ADT design for UAV- VLC systems.

Figure 5c presents the optimized EE contour plot (in bits/J/Hz) for the ADT3 featuring outer-layer-doubling architecture, with the following system parameters: (1) K=3 layers with $n_{1}=1$, $n_{2}=6$, and $n_{3}=12$ LEDs per layer, and rotation angle β=0°; (2) target area radius $r_{c}=3$ m. The joint optimization of the inclination angle (φ) and hover height (*l*) yields a maximum EE of 7.1 bits/J/Hz at the optimal operating point φ=45° and l=2.8 m. Compared to the ADT1 and ADT2 configurations (Figure 5a,b, respectively), the ADT3, which introduces the layer-doubled structure, enhances the EE by 57.7% and 41.9% under identical deployment conditions. These results validate the performance superiority of the layer-doubled design paradigm for UAV-VLC systems.

**Figure 6:**
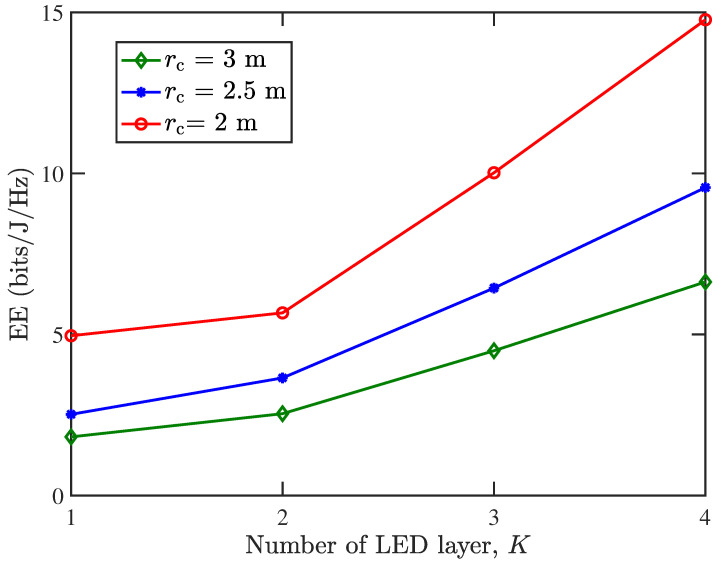
EE (bits/J/Hz) vs. the number of LED layers *K* for ADT1 in the centralized single-UAV VLC system.

Figure 6 presents the EE performance vs. the number of LED layers (*K*) in the ADT1 configuration for the centralized single-UAV VLC system. The baseline case (*K* = 1) corresponds to a conventional single-LED transmitter, while increasing the layer count demonstrates substantial EE improvements regarding different values of $r_{c}$. These results validate the effectiveness of our proposed enhanced ADT structures in enhancing system EE. The observed performance improvements underscore the practical value of this architectural strategy in the design of energy-efficient UAV-VLC systems.

**Figure 7:**
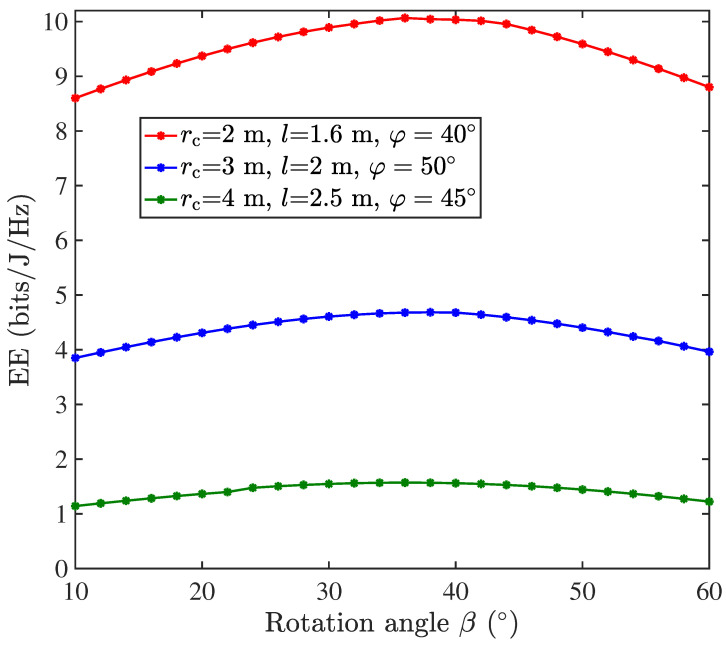
EE (bits/J/Hz) vs. the rotation angle β for ADT2 in the centralized single-UAV VLC system.

The impact of the inter-layer rotation angle β on the performance of the centralized single-UAV VLC system using ADT2 is analyzed in Figure 7. Simulations are conducted under the following parameterization: K=3 layers with $n_{1}=1$, $n_{2}=6$, and $n_{3}=6$ LEDs per layer. It is shown that the system EE follows a concave relationship with β, initially increasing before decreasing to reveal an optimal rotation angle β=36° that maximizes EE. Notably, this optimal angle equals half of the 72° azimuthal spacing between adjacent LEDs within the same layer. The simulations were conducted under optimal UAV operating conditions, including hover height *l* and LED inclination angle φ tuned for the target coverage area of 2 m, 3 m, and 4 m, respectively. These findings indicate that configuring the inter-layer rotation angle at approximately half the intra-layer LED spacing can effectively optimize the EE performance of ADT2-based UAV-VLC systems.

**Figure 8:**
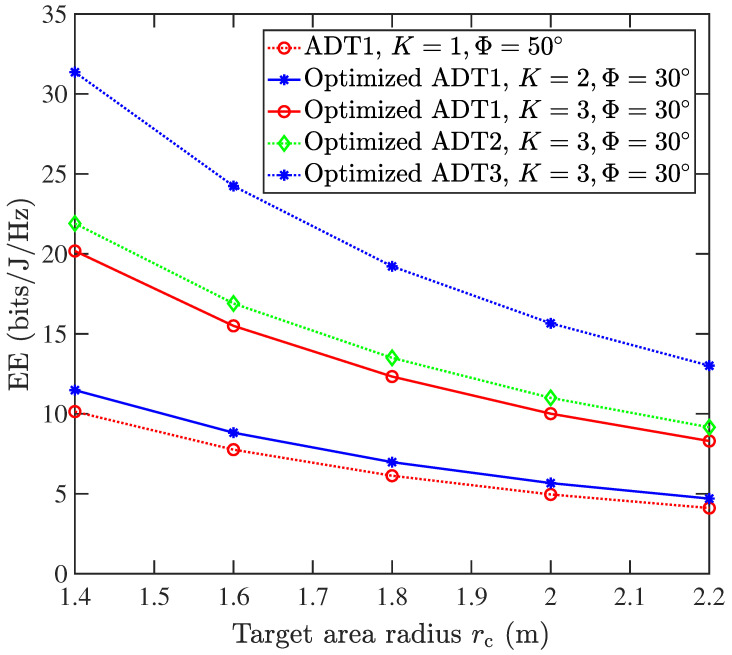
Energy efficiency (bits/J/Hz) vs. the target area radius $r_{c}$ for different ADT configurations in the centralized single-UAV VLC system.

Figure 8 presents a comprehensive performance evaluation of five transmitter architectures in centralized single-UAV VLC systems, revealing three key findings: (1) The EE of the system decreases as the target area radius $r_{c}$ increases; (2) the case of ADT1 with single layer, K=1,Φ=50°, representing a conventional single LED transmitter, exhibits the worst performance; (3) the proposed ADT1 structure with three-layer (*K* = 3) LEDs demonstrates superior performance, achieving an EE of 10.01 bits/J/Hz at $r_{c}=$ 2 m. This represents an EE improvement of 76.4% and 101.8% over the single-layer (*K* = 1, 4.96 bits/J/Hz), and dual-layer (*K* = 2, 5.67 bits/J/Hz) configurations, respectively. This significant improvement highlights the advantage of employing multiple LED layers in the ADT design. (4) Among all three-layer (*K* = 3) ADT structures, both ADT2 and ADT3 consistently outperform ADT1, achieving energy efficiencies of 11 bits/J/Hz and 15.66 bits/J/Hz. These demonstrate EE enhancements of 121.7% and 215.7% over corresponding single LED transmitter (*K* = 1) scenarios. These results validate the benefit of employing inter-layer rotation and layer-doubled design in the ADT configurations.

### 3.2. The Distributed UAV-Array Configuration

**Figure 9:**
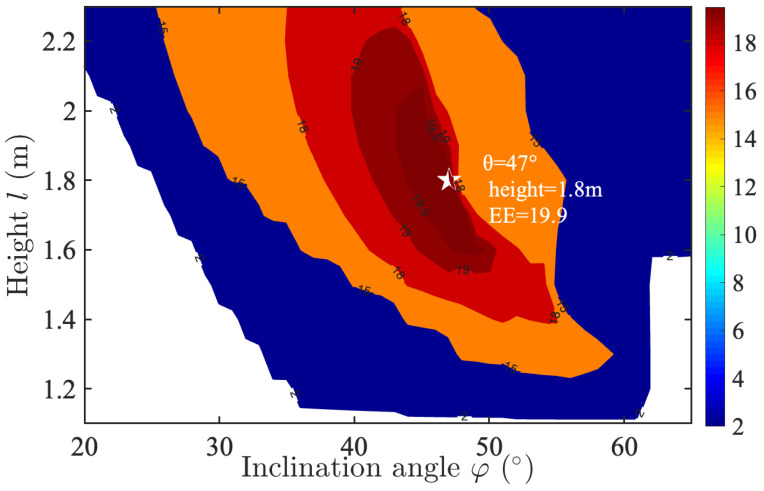
Contour plot of the EE (bits/J/Hz) for ADT2 in the distributed UAV-array system.

In Section 3.2, a comparative performance analysis of multiple ADT configurations implemented in a distributed UAV-array VLC system is conducted. Figure 9 shows the EE contour plot for ADT2, characterizing the coupled effects of LED inclination angle (φ) and UAV hover height (*l*) under the following system parameters: (1) K=3 layers with $n_{1}=1$, $n_{2}=6$, and $n_{3}=6$ LEDs per layer, and inter-layer rotation angle β=36°; (2) target area radius $r_{c}=3.5$ m; (3) the number of drones in the UAV array $N_{\mathrm{UAV}}$ is 4. The analysis identifies an optimal operating point (φ=47°, l=1.8 m) as shown by the white star that achieves a peak EE performance of 19.9 bits/J/Hz, representing an 298% improvement over the corresponding centralized single-UAV configuration with ADT2 in Figure 5b. These results demonstrate the significant advantages of distributed UAV arrays with optimized ADT configurations for energy-efficient VLC systems.

**Figure 10:**
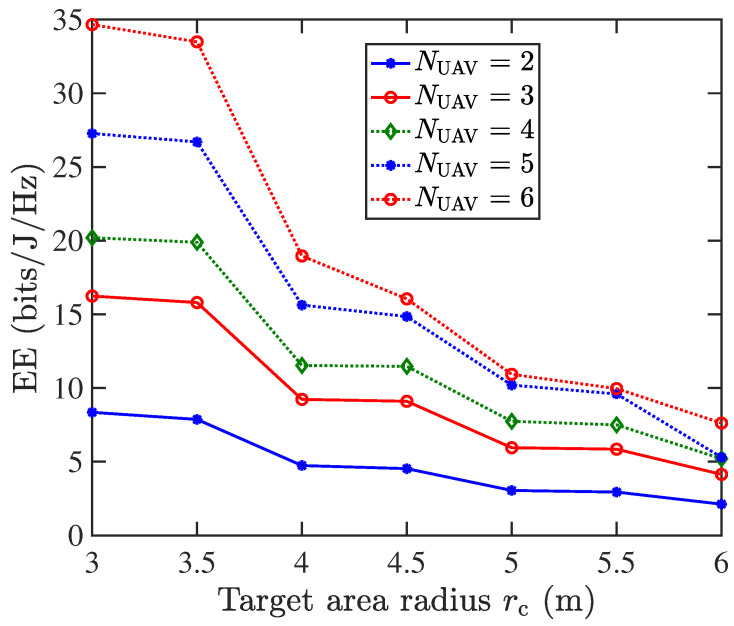
Energy efficiency (bits/J/Hz) vs. the target area radius in the distributed UAV-array VLC system.

Figure 10 analyzes the impact of the target area radius $r_{c}$ and the number of UAVs $N_{\mathrm{UAV}}$ on the performance of the distributed UAV-array VLC system. Simulations are conducted under the following parameterization: (1) the ADT2 structure is used; (2) a three-layer LED configuration (K=3) with $n_{1}=1$, $n_{2}=6$, and $n_{3}=6$ LEDs per layer and an inter-layer rotation angle of β=36°; (3) a fixed UAV formation radius of $r_{\mathrm{UAV}}=1.5$ m; (4) dynamically optimized LED inclination angle φ and UAV hover height *l* for each scenario in Figure 10. The results demonstrate that, for a fixed $N_{\mathrm{UAV}}$, the optimal EE decreases as $r_{c}$ expands, whereas for a given $r_{c}$, the EE improves with increasing $N_{\mathrm{UAV}}$. This indicates that system performance can be enhanced by carefully selecting the target service area size and deploying an optimal number of UAVs to balance communication and illumination efficiency.

## 4. Conclusions

In this paper, we have proposed and evaluated the UAV-array aided VLC systems with enhanced ADTs for joint emergency illumination and communication. Enhanced ADTs with varying LED layers, multiple LEDs per layer, and inter-layer rotation angles are considered. The results indicate that when the UAV hovering altitude and the inclination angle of the ADT side LEDs are jointly optimized, the centralized single-UAV VLC system achieves reduced power consumption while still meeting both illumination and communication requirements. For a coverage radius of 2 m, the proposed ADT1, ADT2, and ADT3 configurations provide energy efficiency improvements of 76.4%, 121.7%, and 215.7%, respectively, compared with the conventional single-LED transmitter. This validate the benefit of employing multi-layer LEDs, inter-layer rotation, and layer-doubled design in the enhanced ADT configurations. Afterwards, the performance of the distributed UAV-array VLC system is analyzed and compared with that of the centralized single-UAV VLC system. The simulation results confirm both the feasibility and the advantage of employing a distributed UAV-array architecture with optimized enhanced ADTs to realize an energy-efficient UAV-VLC system. In particular, when $N_{\mathrm{UAV}}=4$, the distributed UAV-array system attains a peak energy efficiency of 19.9 bits/J/Hz, which corresponds to a 298% improvement over the centralized single-UAV case. Moreover, the results show that the energy efficiency of the distributed UAV array system increases further as $N_{\mathrm{UAV}}$ grows. Therefore, the centralized single-UAV system is simpler and sufficient for smaller areas, while the distributed UAV array is more complex to control but delivers far superior energy efficiency and scalability, making it the promising candidate for future emergency illumination and communication networks. In future studies, we plan to incorporate atmospheric attenuation, UAV vibrations, and other practical real-world considerations to further enhance the system model.

## Figures and Tables

**Table 1:** Simulation parameters.

Parameter	Value
Gain of filter $g_{f}$	0.9
FOV of optical concentrator Ψ	65°
Refractive index of optical concentrator *n*	1.5
Active receiving area of PD *A*	1 cm^2^
Noise PSD	$1\times 10^{-22}$ A^2^/Hz
Photoelectric conversion coefficient	1 W/A
Responsivity	0.8 A/W
Signal bandwidth	10 MHz
Semi-angle at half power of the LED Φ	30°
Channel capacity threshold $C_{\mathrm{th}}$	1 bit/s/Hz
Illuminance threshold $\kappa_{\mathrm{th}}$	$5\times 10^{-4}$ Lx

## Data Availability

The data of this study are available from the corresponding author upon request.
